# Recipient‐Donor Sex Constellation on Outcomes After Liver Transplant for Hepatocellular Carcinoma: An External Validation Analysis

**DOI:** 10.1111/liv.70123

**Published:** 2025-05-05

**Authors:** Lauren E. Matevish, Amit G. Singal, Gonzalo Sapisochin, Nathanael Raschzok, Nicole Rich, Arjmand Mufti, Parsia A. Vagefi, Madhukar S. Patel

**Affiliations:** ^1^ Division of Surgical Transplantation, Department of Surgery University of Texas Southwestern Medical Center Dallas Texas USA; ^2^ Division of Digestive and Liver Disease, Department of Medicine University of Texas Southwestern Medical Center Dallas Texas USA; ^3^ HBP and Multi‐Organ Transplant Program, Division of General Surgery University Health Network, University of Toronto Toronto Ontario Canada; ^4^ Department of Surgery Campus Virchow Klinikum, Campus Charité Mitte, Charité—Universitätsmedizin Berlin Berlin Germany; ^5^ Berlin Institute of Health at Charité—Universitätsmedizin Berlin, BIH Academy, Clinician Scientist Program Berlin Germany

**Keywords:** hepatocellular carcinoma, liver transplant, patient mortality, recipient/donor sex

**Abbreviations**
AFP: alpha‐fetoprotein
BMI: body mass index
FRFD: female recipient‐female donor
FRMD: female recipient‐male donor
HCC: hepatocellular carcinoma
LRT: locoregional treatment
LT: liver transplant
MRFD: male recipient‐female donor
MRMD: male recipient‐male donor
OS: overall survival
RDSC: Recipient‐Donor Sex Constellation
TACE: transarterial chemoembolization
UNOS: United Network for Organ Sharing

To the Editor,

1

It was with great interest that we read the recent article published in *Liver International*, ‘Recipient‐Donor Sex Constellation in Liver Transplantation for Hepatocellular Carcinoma—An ELTR Study,’ by Magyar et al. from the European Liver and Intestine Transplant Association [1]. While several associations between sex and post‐transplant outcomes have been previously noted in the literature [2, 3, 4], the finding in this current report demonstrates a novel association of improved survival rates in women with hepatocellular carcinoma (HCC) undergoing liver transplant (LT) with a graft from a male donor. We sought to determine whether this association, identified in a European population, persists in a sample from the United States.

Using the UNOS Standard Transplant and Analysis Research files, we identified all patients who underwent deceased donor LT for a primary or secondary diagnosis of HCC from 2012 to 2024 in the United States. Patients were stratified by recipient‐donor sex constellation (RDSC), as female recipient‐female donor (FRFD), female recipient‐male donor (FRMD), male recipient‐female donor (MRFD) or male recipient‐male donor (MRMD). Patients with incomplete explant pathology or HCC treatment data were excluded. Cox proportional hazards regression modelling was used to compare overall survival (OS), adjusting initially for factors in the European model as well as several a priori additions; these covariates included liver disease aetiology (metabolic dysfunction‐associated steatotic liver disease, hepatitis B virus, alcohol‐associated liver disease), age difference between donor and recipient, donor and recipient body mass indices, donor type (donor after circulatory death or donor after brain death), Risk Estimation of Tumour Recurrence After Transplant (RETREAT) score [5], worst tumour differentiation, and total number of pre‐transplant locoregional treatments (LRT). A Fine‐Grey competing risk regression model was subsequently performed for recurrence‐related death, with other causes of death (e.g., cardiovascular, stroke, graft failure, etc.) as competing outcomes. All analyses were performed using Stata 18 (StataCorp, College Station, TX), and a *p* < 0.05 was considered statistically significant. The UT Southwestern IRB determined the study was exempt.

A total of 28 313 adult patients who underwent first‐time LT for a primary or secondary diagnosis of HCC were identified; 14 685 lacked complete explant data and were excluded. Of the remaining 13 628 LT recipients, the distribution of RDSC was MRMD 49.2%, MRFD 27.7%, FRMD 11.2%, and FRFD 11.9%. Median age was 63.0 years (IQR 58.0–67.0 years) and 76.9% were men. RDSC groups differed significantly in recipient age and BMI, liver disease aetiology, pre‐transplant ascites, and both donor age and BMI. Female recipient groups had a higher proportion of patients within Milan criteria on explant, while less than 60% of male recipients were within Milan (Table 1). Furthermore, tumour burden differed between groups, with male recipients having a higher median number of tumours, greater total tumour volume, higher rates of vascular invasion, and poorer differentiation. Pre‐transplant LRT modalities were similar across RDSC groups. Patients had a median of 1 treatment (IQR 1–2), and transarterial chemoembolization (TACE) was the most common LRT, administered to 61.6% of patients; 24.4% of patients received multimodal therapy (i.e., two or more different LRT types).

**TABLE 1:** Comparison of HCC tumour and treatment factors amongst RDSC groups.

	FRFD *N* = 1620 (11.9%)	FRMD *N* = 1533 (11.2%)	MRFD *N* = 3770 (27.7%)	MRMD *N* = 6705 (49.2%)	*p*
AFP	9 [4–25]	8 [4–25]	7 [4–18]	7 [4–17]	**< 0.001**
Total tumours	1 [1–2]	1 [1–2]	2 [1–3]	2 [1–3]	**< 0.001**
1	813 (55.3%)	743 (54.5%)	1656 (46.9%)	2906 (46.2%)	**< 0.001**
2	345 (23.5%)	285 (20.9%)	885 (25.1%)	1513 (24.1%)	
3	138 (9.4%)	162 (11.9%)	421 (11.9%)	814 (12.9%)	
4	65 (4.4%)	73 (5.4%)	235 (6.7%)	430 (6.8%)	
5	51 (3.5%)	33 (2.4%)	140 (4.0%)	265 (4.2%)	
6+	52 (3.5%)	61 (4.5%)	178 (5.0%)	327 (5.2%)	
Infiltrative	6 (0.4%)	6 (0.4%)	15 (0.4%)	35 (0.6%)	
Largest tumour size (cm)	2.5 [1.7–3.8]	2.5 [1.7–3.7]	2.8 [1.9–4.0]	2.8 [2.0–4.0]	**< 0.001**
Total tumour volume (cm^3^)	8.2 [1.8–28.7]	8.2 [1.5–26.5]	12.4 [3.1–36.1]	12.8 [3.1–35.7]	**< 0.001**
Vascular invasion					
None	1311 (89.2%)	1188 (87.2%)	2986 (84.6%)	5309 (84.4%)	**< 0.001**
Microvascular	142 (9.7%)	156 (11.4%)	471 (13.3%)	841 (13.4%)	
Macrovascular	17 (1.2%)	19 (1.4%)	73 (2.1%)	140 (2.2%)	
Worst tumour differentiation					
Complete tumour necrosis	427 (29.0%)	365 (26.8%)	824 (23.3%)	1383 (22.0%)	**< 0.001**
Well	321 (21.8%)	299 (21.9%)	709 (20.1%)	1312 (20.9%)	
Moderate	633 (43.1%)	614 (45.0%)	1725 (48.9%)	3138 (49.9%)	
Poor	89 (6.1%)	85 (6.2%)	272 (7.7%)	457 (7.3%)	
Within Milan	65.3%	61.6%	58.8%	59.0%	**< 0.001**
Surgical resection	1.3%	1.2%	1.9%	1.6%	0.21
Locoregional treatments					
TACE	59.7%	61.3%	63.0%	61.5%	0.13
TARE	17.6%	19.0%	17.9%	17.7%	0.69
RFA	5.0%	5.6%	5.4%	5.2%	0.89
Cryoablation	0.2%	0.1%	0.0%	0.1%	0.26
Chemical ablation	2.9%	3.8%	4.0%	3.8%	0.27
Thermal ablation	25.9%	24.5%	26.5%	27.5%	0.08
SBRT	4.0%	4.7%	3.6%	4.1%	0.28
Total treatments	1 [1–2]	1 [1–2]	1 [1–2]	1 [1–2]	0.26
Multimodal treatment	22.0%	23.9%	24.8%	24.8%	0.10

Abbreviations: FRFD, female recipient female donor; FRMD, female recipient male donor; HCC, hepatocellular carcinoma; MRFD, male recipient female donor; MRMD, male recipient male donor; RDSC, recipient donor sex constellation; RFA, radiofrequency ablation; SBRT, stereotactic body radiation therapy; TACE, transarterial chemoembolization; TARE, transarterial radioembolization. Bolded values if *p* < 0.05.

Over a median follow‐up of 57.6 months (IQR 23.0–91.9 months), death was observed in 24.0% of patients. Median OS was 57.9 months (IQR 23.1–92.7 months), with a 5‐year OS of 83.6%. There were no significant differences in unadjusted 5‐year OS rates between RDSC groups (MRMD 83.8%, MRFD 82.9%, FRMD 84.3%, FRFD 83.8%; *p* = 0.54), nor in median patient survival time (i.e., from LT to registered death) (MRMD 58.0 months, MRFD 59.2 months, FRMD 50.7 months, FRFD 57.1 months; *p* = 0.74). However, on multivariable analysis, FRMD pairing was significantly associated with a 16% reduction in the risk of death (FRFD as reference: FRMD aHR 0.84 [0.71–0.99], MRFD aHR 1.05 [0.92, 1.20], MRMD aHR 1.00 [0.89, 1.14]). Adjusted Kaplan–Meier survival rates were similar across RDSC groups at 5 years (MRMD 86.4%, MRFD 75.1%, FRMD 86.3%, FRFD 77.0%) with greater disparities seen at 10 years (MRMD 71.4%, MRFD 50.9%, FRMD 78.5%, FRFD 55.3%) (Figure 1A). No significant impact of RDSC group was seen on graft survival (FRFD as reference: FRMD aHR 0.93 [0.80–1.10], MRFD aHR 1.10 [0.97, 1.25], MRMD aHR 1.05 [0.93, 1.18]).

**FIGURE 1:**
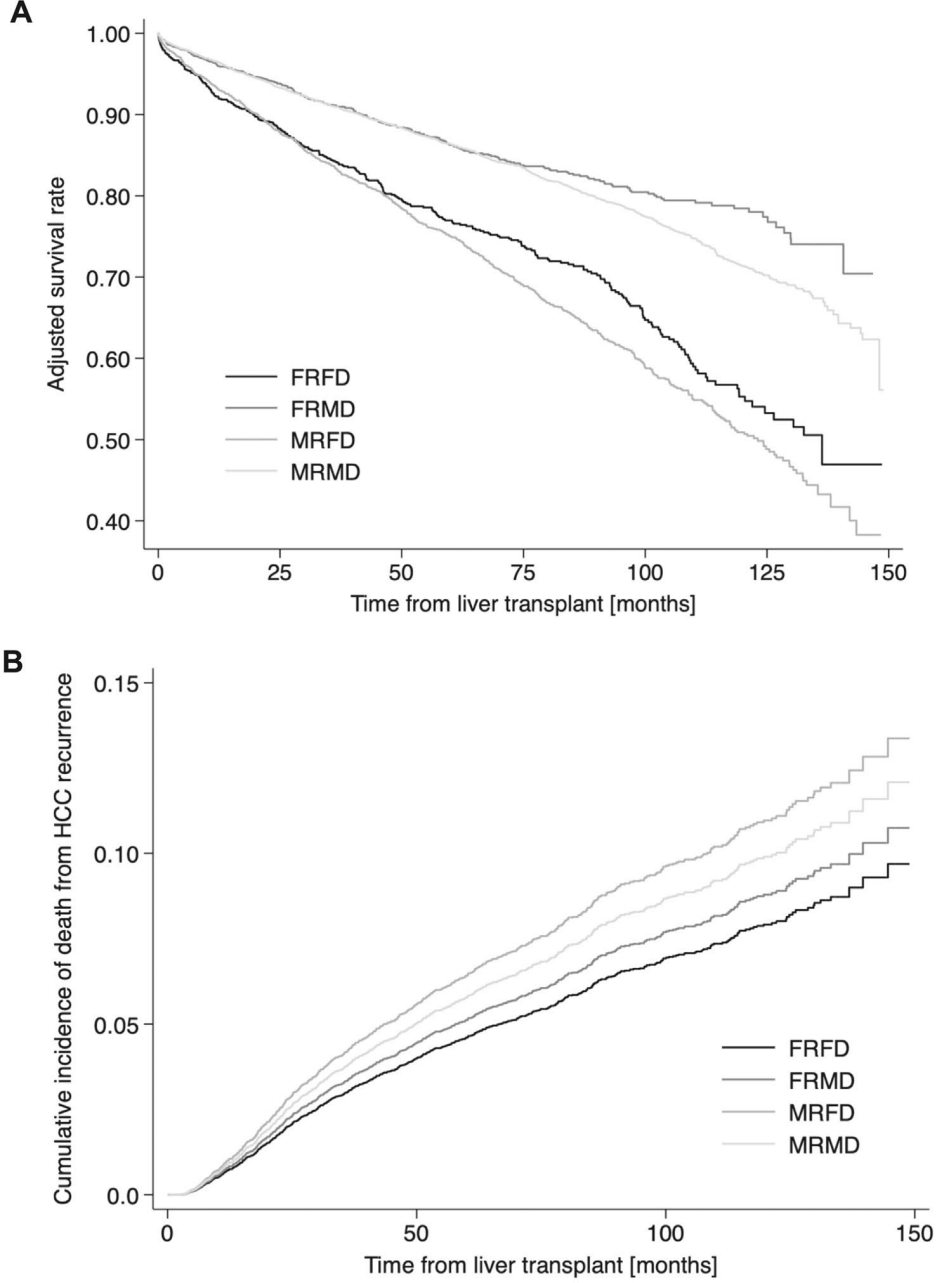
(A) Adjusted Kaplan–Meier survival analysis of patients with HCC after liver transplant. (B) Adjusted cumulative incidence curve of the rate of death due to HCC recurrence by RDSC group. Curves adjusted for MASLD, hepatitis B virus, alcohol‐associated liver disease, age difference between donor and recipient, donor and recipient BMI, donor type (DCD or DBD), RETREAT score, worst tumor differentiation, and total number of pre‐transplant LRTs.

Recurrent malignancy was the most common cause of death across RDSC groups, with incidence rates significantly higher in the MRFD group (FRFD 4.9% vs. FRMD 6.2%, MRFD 8.3%, MRMD 7.0%; *p* < 0.001). This represented 40.8% of all registered mortalities in the MRFD population compared to 27.2% in the FRFD group. Furthermore, the MRFD constellation was associated with an increase in the cumulative incidence of death due to HCC recurrence (sHR 1.41 [1.08–1.83]) (Figure 1B). Increasing RETREAT score (sHR 1.34 [1.27–1.41]), tumour histologic differentiation (well differentiated sHR 1.60 [1.28–2.00]; poorly differentiated sHR 3.57 [2.72–4.68]), and total number of LRTs (sHR 1.10 [1.05–1.16]) were also noted to be significant in the model. To further explore the association between female donor and death from HCC recurrence, we performed subgroup analysis by pre‐ (preM, age ≤ 40 years) vs. post‐menopausal (postM, age ≥ 55 years) status. On competing risk analysis, no difference in death from HCC recurrence was observed based on menopausal status (FRFD preM as reference; FRFD postM sHR 0.93 [0.46, 1.83], MRFD preM sHR 1.15 [0.76, 1.76], MRFD postM sHR 1.35 [0.73, 2.50]).

In our study of U.S. LT recipients with HCC, we found a lower mortality risk in the FRMD constellation, consistent with results from the European cohort [1]. The external validation of this finding suggests that there are likely biologic factors at play rather than environmental. While the prior study did not find a significant difference across groups for recurrence‐related mortality, we found higher odds in the MRFD constellation on competing risk regression. This finding is concordant with known higher rates of HCC recurrence in men, though it is perhaps unexpected that a female donor graft would result in worse outcomes than the MRMD group. These results show that female recipients appear to have superior OS undergoing LT with a male donor graft, while male recipients transplanted with male donor grafts benefit from improved HCC recurrence‐specific mortality outcomes. LT with selection of a male donor may be more feasible in patients with a living donor option, though in the US, nearly 95% of all LTs are performed using a deceased donor graft [6]. While about two‐thirds of LTs use male donors [6], anthropomorphic mismatch often necessitates use of female donors for female recipients. A better understanding of the underlying biologic and hormonal pathways at play in the recurrence of HCC could help drive targeted therapies for tertiary prevention.

We acknowledge several limitations with our study. First, although our study population mirrors the HCC sex discrepancy seen in the general population, the smaller number of female recipients creates a possibility of type 2 statistical error, particularly when evaluating infrequent outcomes. Indeed, in the subgroup analysis of pre‐ and post‐menopausal donors, the lack of significance may potentially be related to an underpowered analysis. Additionally, due to limited availability of pathologic explant data, only patient records from 2012 and after were included, truncating available data points. However, with changes in HCC treatment over the past decade, a contemporary analysis ensures that the results are more applicable to current populations. Finally, the UNOS data, as a retrospectively collected database, is prone to measurement bias and residual confounding. Specifically, recipient factors such as significant medical comorbidities, immunosuppression regimen and adherence, and substance use or recidivism are lacking, all of which may contribute to mortality.

In summary, we found that RDSC was independently associated with mortality in a U.S. cohort, with improved OS in the FRMD group, validating the results previously published by Magyar et al. using European data. Further, we found MRFD was independently associated with death from HCC recurrence. Further work is needed to elucidate mechanisms through which sex, both recipient and donor, can impact post‐LT survival and HCC recurrence.

## Author Contributions

**Lauren E. Matevish:** contributed to the conception, design, analysis, and interpretation of data, and drafting the manuscript. **Amit G. Singal:** contributed to the conception, design, and interpretation of data, as well as critical revisions. **Gonzalo Sapisochin, Nathanael Raschzok, Nicole Rich, Arjmand Mufti, Parsia A. Vagefi:** contributed to the interpretation of data and critical revisions. **Madhukar S. Patel:** contributed to the conception, design, analysis, and interpretation of data, as well as critical revisions.

## Conflicts of Interest

Parsia A. Vagefi is a consultant for TransMedics National OCS Steering Committee and a member of the Davita Innovation Advisory Board. Amit G. Singal has served as a consultant or on advisory boards for Genentech, AstraZeneca, Eisai, Bayer, Exelixis, Merck, Boston Scientific, Sirtex, HistoSonics, FujiFilm Medical Sciences, Exact Sciences, Roche, Glycotest, Abbott, Freenome, and GRAIL.

## Data Availability

The data that support the findings of this study are openly available in the UNOS Standard Transplant and Analysis Research files at https://optn.transplant.hrsa.gov/data/view‐data‐reports/request‐data/.
